# The Impact of Water Exchange on Estimates of Myocardial Blood Flow and Extracellular Volume Fraction Using Dynamic Contrast‐Enhanced MRI in Patients Treated for Severe Aortic Stenosis

**DOI:** 10.1002/mrm.70571

**Published:** 2026-08-20

**Authors:** Alex Makins, John D. Biglands, Noor Sharrack, Peter Kellman, Sven Plein, David L. Buckley

**Affiliations:** ^1^ Biomedical Imaging, Leeds Institute of Cardiovascular and Metabolic Medicine University of Leeds Leeds UK; ^2^ Department of Medical Physics & Engineering Leeds Teaching Hospitals NHS Trust Leeds UK; ^3^ National Heart, Lung, and Blood Institute National Institutes of Health Bethesda Maryland USA

**Keywords:** aortic stenosis, extracellular volume, myocardial blood flow, water exchange

## Abstract

**Purpose:**

To investigate the impact of intra/extracellular water exchange (WX) on quantitative dynamic contrast‐enhanced (DCE) MRI in patients with severe aortic stenosis (AS). The aims were to compare low‐dose (WX insensitive) and high‐dose (WX sensitive) DCE‐MRI measurements, and to compare data acquired before and after aortic valve replacement (AVR) to test whether DCE‐MRI was able to detect changes in myocardial extracellular volume fraction (ECV).

**Methods:**

Twenty‐five patients with severe AS were studied at 3 T before and 6 months after AVR. DCE‐MRI was performed at rest twice using a low contrast agent dose (0.05 mmol/kg gadobutrol) followed by a high‐dose (0.1 mmol/kg gadobutrol). Estimates of myocardial blood flow (MBF) and ECV were obtained using models which assume instantaneous WX (fast exchange [FX]) and which incorporate WX (a two‐site exchange [2SX] model). ECV estimates were also made using T1 maps acquired before and after contrast administration, both assuming FX and incorporating WX.

**Results:**

FX model estimates of MBF and ECV obtained before AVR decreased from low‐ to high‐dose contrast consistent with the effect of limited WX. This decrease was partially corrected using a 2SX model. However, 2SX model fits were preferred over FX fits in only 2 of 24 high‐dose data sets (*F*‐test). ECV estimates made using DCE‐MRI increased after AVR consistent with changes seen using T1 maps, but the ECV estimates were lower.

**Conclusion:**

Estimates of MBF using DCE‐MRI showed negligible sensitivity to WX. DCE‐MRI‐based ECV estimates changed post‐AVR but were systematically lower than those made using T1 maps.

## 1 Introduction

It has long been recognized that water exchange (WX) between tissue spaces can impact estimates of myocardial blood flow (MBF) made using first‐pass perfusion techniques, also known as dynamic contrast‐enhanced (DCE) MRI [1]. At low contrast agent doses, WX effects are small because the difference in T1 between those tissue spaces remains small, and previous attempts to measure WX alongside MBF using conventional low‐dose DCE‐MRI have failed [2]. Conversely, in clinical routine, extracellular volume fraction (ECV) in the myocardium is regularly measured using T1 maps acquired before and after higher contrast agent doses [3] and this can lead to ECV underestimates if WX between the cells (cardiomyocytes) and the extracellular space is ignored. However, when WX is modeled appropriately, ECV can be measured accurately [4, 5].

Gadolinium based contrast agents enhance the MR signal by reducing the T1 of nearby water molecules. Although the contrast agents themselves cannot pass through healthy cell walls, water molecules that they have interacted with can. Therefore, the exchange of water between extracellular and intracellular spaces must be considered when modeling contrast‐enhanced data [6]. A common assumption made when making T1 map‐based contrast enhanced MRI measurements is that the exchange of water between the cardiomyocytes and the extracellular space is instantaneous, known as fast exchange (FX). If the FX assumption is true, then the two spaces can be treated as one and estimates of ECV can be made using a linear model (LM), which assumes a single myocardial relaxation rate (1/T1) which changes linearly with changes in 1/T1 in the blood [7]. However, previous studies have demonstrated that there is a limited rate of WX between these spaces in the myocardium and failure to accommodate this leads to underestimates in ECV [4, 5]. These underestimates are more pronounced at higher contrast agent concentrations where there is a bigger difference between T1 inside and T1 outside the cell. The LM can be replaced with a simplified version of a two‐site exchange (2SX) model known as a “shutter speed” (SS) model, which largely accounts for the effects of limited WX, producing more accurate ECV estimates [8].

DCE‐MRI is conventionally used to assess MBF only, but it also provides an alternative method for the measurement of ECV [9]. Here the signal change in the myocardium is measured over time and a tracer kinetic model is used to describe the movement of the contrast agent into the tissue's extracellular space [10]. More complex tracer kinetic models can estimate additional physiological parameters [11], however they can be difficult to fit and can suffer from overfitting (i.e., they contain more parameters than are needed to describe the data) [12, 13]. It has also been shown that limited WX between cell and extracellular space impacts estimates of tracer kinetic parameters used in DCE‐MRI [1, 2]. Here the use of a full 2SX model is required to account for the effects of WX on rapid T1‐weighted images (rather than the SS approximation which describes the impact on T1 measurements) and this can be incorporated into the tracer kinetic model to analyze DCE‐MRI data [14, 15].

Aortic stenosis (AS) is a common heart valve condition which can lead to chronic pressure overload of the left ventricle (LV), giving rise to ventricular hypertrophy and the formation of diffuse or focal myocardial fibrosis [16]. In severe cases, if left untreated, this can ultimately lead to LV dysfunction and heart failure [6]. However, in as few as 2 months following aortic valve replacement (AVR), there is a reversal of myocardial cell hypertrophy and an increase in ECV [17]. By 6 months post‐AVR the increase in ECV and reduction in cell hypertrophy is sufficient to see an increase in the rate of intra/extracellular WX [18].

The aims of this study were twofold. Firstly, to determine if the effects of limited intra/extracellular WX could be detected in a DCE‐MRI experiment if a high dose of contrast agent was administered. We hypothesized that a high dose would increase the impact of WX on estimates of MBF and ECV. Secondly, to demonstrate that changes in ECV due to the reversal of myocardial hypertrophy following AVR, previously demonstrated using T1 mapping experiments [18], could also be measured using DCE‐MRI. We compared estimates of ECV made using DCE‐MRI with estimates made using T1 maps in patients with AS scanned before and 6 months after AVR.

## 2 Methods

### 2.1 Patient Population

Twenty‐five patients with severe AS referred for AVR surgery were recruited between January and August 2022. Eligibility criteria were detailed in a previous publication [5]. Patients underwent two visits during which MRI data were collected. Visit one was undertaken prior to AVR and visit two took place 6 months post‐AVR [18].

The study protocol was approved by the institutional research ethics committee and complied with the Declaration of Helsinki; all patients gave written informed consent for their participation (REC reference 18/YH/0169).

### 2.2 Cardiovascular Magnetic Resonance Imaging

MRI was performed at 3 T (Magnetom Prisma; Siemens, Erlangen, Germany). Two sets of DCE‐MRI data were acquired at rest, first following a 0.05 mmol/kg bolus of gadobutrol (Gadovist; Bayer AG) injected at 4 mL/s (injection‐1) and secondly, ∼8 min later, following a further 0.1 mmol/kg infusion of gadobutrol delivered at a lower rate of 0.5 mL/s to avoid saturation of signal in the LV blood pool (injection‐2). T1 measurements were made (a) before contrast, (b) 4 min after injection‐1 and (c) 4 min, (d) 10 min, and (e) 30 min after injection‐2 (Figure 1).

**FIGURE 1:**
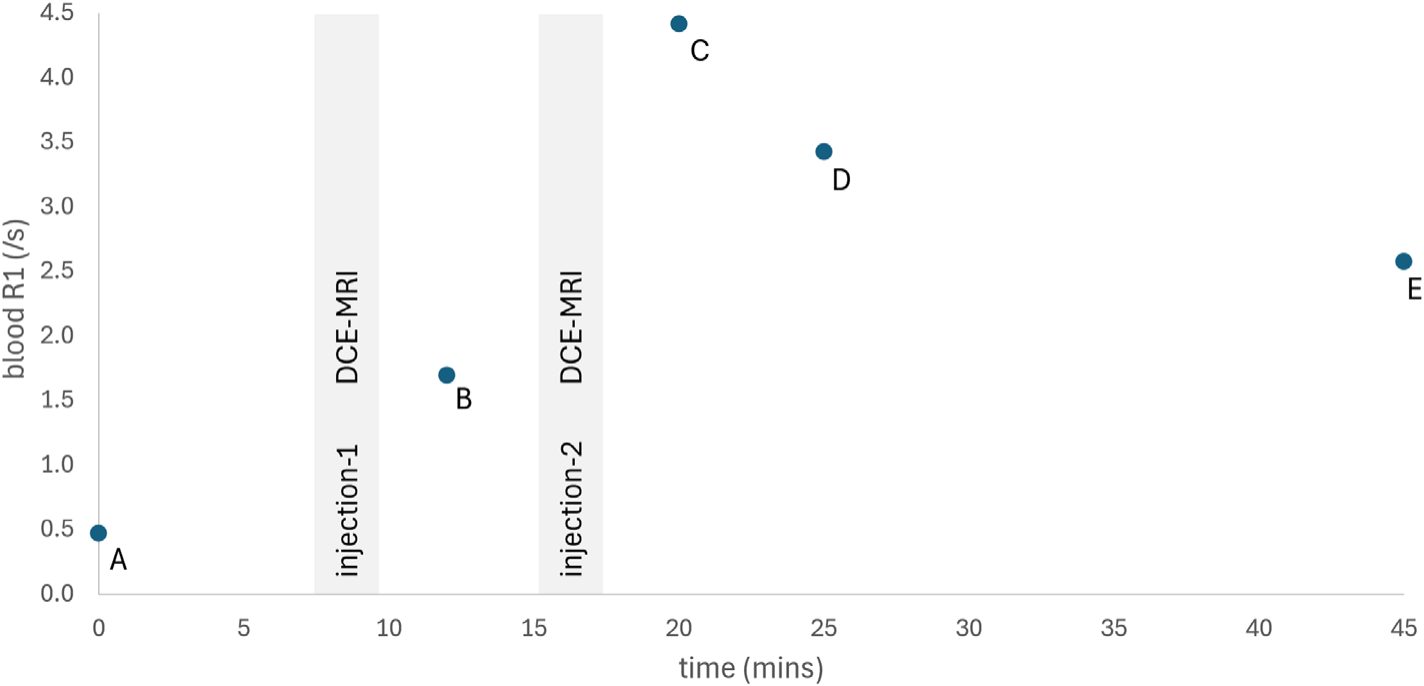
Image acquisition protocol (data from one patient shown as an exemplar). DCE‐MRI data were acquired following the bolus injection of 0.05 mmol/kg gadobutrol (injection‐1) and following the infusion of 0.1 mmol/kg of gadobutrol (injection‐2). These were accompanied by T1 measurements made (A) pre contrast, (B) 4 min post injection‐1, (C) 4 min, (D) 10 min, and (E) 30 min post injection‐2. The shaded areas illustrate the period over which the DCE‐MRI data were acquired.

For both DCE‐MRI and T1 mapping, three 8‐mm short‐axis myocardial slice locations were used, distributed equally using the “3 of 5” method to provide reproducible basal, mid, and apical slice locations [19]. The DCE‐MRI data were acquired over 120 heart beats using a dual sequence approach [20] combined with selected features of an automatic in‐line perfusion mapping methodology [21]. Low spatial resolution, dual echo, saturation recovery FLASH images were acquired in a single basal slice immediately after the R‐wave trigger to characterize the arterial input function (AIF). The dual echoes were used to correct the measured AIF for T2* effects. Subsequently, higher spatial resolution saturation recovery FLASH images were acquired to visualize the myocardium at the three short‐axis slice locations. T1 maps were acquired using a multiparametric saturation recovery single‐shot acquisition (mSASHA) [22], using six images of variable saturation recovery time, and two images with different T2 preparations to generate both T1 and T2 maps. Detailed acquisition parameters for the DCE and T1 mapping are given in Table 1. In addition to these acquisitions, a standard imaging protocol was used consisting of a short axis cine stack covering the entire LV, long axis cine images of the LV (two‐chamber, three‐chamber, and four‐chamber), and short and long axis late gadolinium enhancement (LGE) imaging (performed 5–15 min after the cumulative dose of 0.15 mmol/kg gadobutrol) [23].

**TABLE 1:** Acquisition parameters for the T1 mapping and perfusion sequences.

	mSASHA sequence	Perfusion sequence (FLASH)	AIF sequence (FLASH)
Slices	3 (basal, mid and apical)	3 (basal, mid and apical)	1 basal slice
Slice width	8 mm	8 mm	10 mm
FOV	360 × 270 mm	360 × 270 mm	360 × 270 mm
Matrix	256 × 192	192 × 111	64 × 34
Voxel size	1.4 × 1.4 mm	1.9 × 2.4 mm	5.6 × 8 mm
TR/TE	2.9/1.26 ms	2.1/1.04 ms	2.45/0.76 and 1.76 ms
TS	1 × no preparation 6 × 550 ms pre‐contrast 6 × 300 ms post‐contrast	110 ms	23.8 ms
Flip angle *α*	Variable up to 100°	14°	5°
Acceleration	GRAPPA 2	T‐PAT 3	T‐PAT 2

### 2.3 MRI Data Analysis

In‐line post processing of the AIF DCE‐MRI data included motion correction, proton density correction, and contrast concentration conversion [21]. For the myocardial perfusion data, only motion correction was performed as the in‐line contrast concentration conversion implicitly assumes FX.

Myocardial contouring of the DCE and T1 data was undertaken using cvi42 software (Circle Cardiovascular Imaging Inc., Calgary, Canada, release number 5.9.4). For T1 mapping, short axis mid‐ventricular T1 maps were manually contoured at the endocardial and epicardial borders, excluding papillary muscles and any wall edge artifacts. Partial volume effects of blood were minimized by setting an automatic offset of 10% from the borders. T1 of the myocardium and LV blood pool, together with the patient's hematocrit (Hct), was used to estimate ECV [3, 5]. Hct measurement was not performed for this study specifically; we used the closest result measured during the patient's routine clinical visits. Linear model ECV (ECV(LM)) was estimated using the T1 maps obtained before contrast and at 10 min after injection‐2, while shutter speed ECV estimates (ECV(SS)) were made using all 5 T1 maps [5].

Myocardial contours were drawn on the DCE‐MRI data using the same approach used for the T1 maps starting with images acquired at the time point with maximal contrast between the blood pool and myocardial wall. These contours were then copied to all time points and manually checked. Any misalignment due to failure in the motion correction was then corrected by manual rigid translation of the contours. The blood pool was automatically segmented from the low spatial resolution DCE‐MRI data and used to generate an AIF [20].

The myocardial signals from the DCE‐MRI data were analyzed using software developed in Matlab. Modeling was performed using methods detailed by Buckley et al. [14] which assume distribution of the contrast agent according to a single compartment model [24]. The single compartment represents the extracellular fluid volume, while WX takes place between two “sites” (the cells and the extracellular fluid [6]). To help differentiate between WX and contrast agent exchange, we use the terms sites and compartments, respectively. Contrast agent uptake is described by two parameters, MBF and ECV. WX is also described by two parameters, ECV and the intracellular lifetime of water (τ_ic_). The three parameters can, in principle, be simultaneously obtained by fitting the combination of single compartment contrast agent and two‐site WX model to the myocardial data [14, 15]. If WX is assumed to be in the FX limit, then only two parameters (MBF and ECV) can be estimated (τ_ic_ is zero under this assumption). In order to perform these analyses, we require an AIF, the baseline myocardial T1 (obtained from the pre‐contrast T1 map for injection‐1 and the 4‐min post‐injection‐1 T1 map for injection‐2), and equations for the signal evolution of a SR‐FLASH sequence [25]. Full details of all the equations used to analyze the DCE‐MRI data are provided in the “Theory” section of [14].

We compared estimates of MBF and ECV made following injection‐1 and injection‐2. As we expect the effect of limited WX to be more prominent at higher contrast agent concentrations, we anticipated a decrease in estimates of both parameters following injection‐2. To confirm that any decrease could be explained by limited WX, we also compared the quality of the FX and 2SX fits using an *F*‐test (the models are nested). In patients with AS who have not yet been treated, the cardiomyocytes are large, WX is slow and so the effects of WX are seen more easily [18]. Therefore, to maximize the chances of observing the effects of limited WX, the pre‐AVR data were used in these comparisons. To assess whether changes in ECV following AVR were observable in the DCE‐MRI data, the ECV(DCE) estimates before and after AVR were obtained (using the FX model only, see Section 4) then compared to the ECV estimates from the T1 analysis (ECV(LM) and ECV(SS)) both pre‐ and post‐AVR.

### 2.4 Statistical Analysis

Statistical analyses were carried out using R Studio version 2022.07.2+576 (R Foundation for Statistical Computing, Vienna, Austria). Normality of the data was checked using the Shapiro–Wilk test. Continuous normally distributed data were expressed as mean ± SD, and continuous non‐normally distributed data as median (range). Comparison between quantitative variables was performed by paired‐sample parametric (Student's *t*‐test) or non‐parametric (Wilcoxon signed‐rank) statistical tests, as appropriate. *p* < 0.05 was considered significant.

## 3 Results

### 3.1 Patient Population

Of the 25 patients recruited, all completed the first visit (pre‐AVR) and 20 completed the second visit (post‐AVR) [18]. Patients had an average age of 68 ± 7 years and 14 were male. Data were acquired before and 174 (157–267) days after AVR.

### 3.2 The Effect of Water Exchange on Tracer Kinetic Parameters

MBF and ECV estimates were successfully obtained from 25 pre‐AVR DCE‐MRI data sets acquired following injection‐1 and 24 data sets acquired following injection‐2 (one data set was not acquired in error). Example data sets are shown in Figure 2.

**FIGURE 2:**
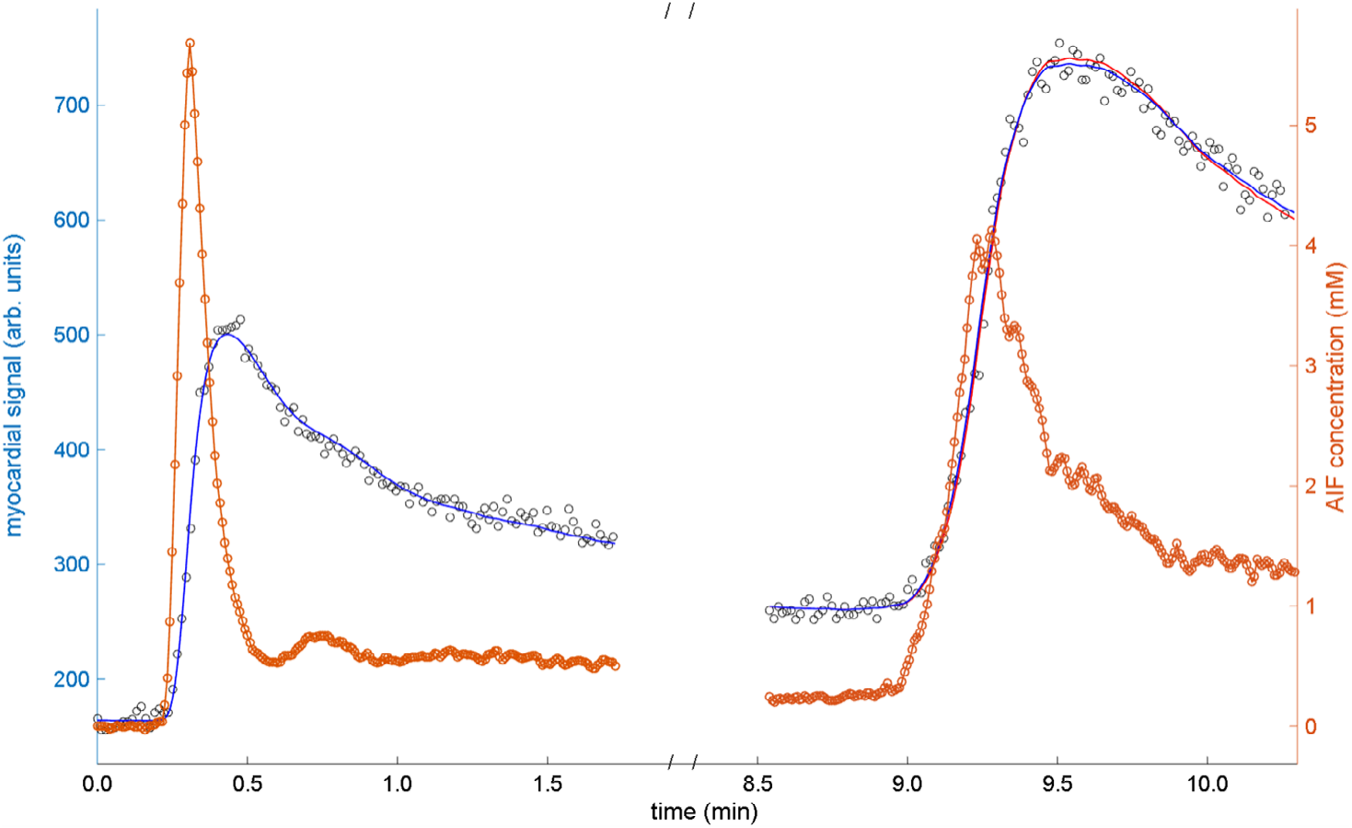
Plots of myocardial signal intensity [a.u.] (open symbols—left hand axis), AIF concentration [mM] (orange plots—right hand axis), and fitted tracer kinetic models (solid lines). The red fit illustrates the FX model and the blue illustrates 2SX (these are indistinguishable following injection‐1).

Estimates of MBF, ECV and τ_ic_ obtained from the DCE‐MRI data acquired following injection‐1 and injection‐2 are summarized in Table 2. Mean MBF estimated using an FX model decreased from injection‐1 to injection‐2 (*p* = 0.02; Figure 3 left panel). A similar effect was seen with mean ECV (*p* = 0.002; Figure 4 left panel). When the injection‐2 data were analyzed using a 2SX model, MBF and ECV estimates increased and, while they remained below the injection‐1 FX estimates, there were no longer significant differences between the injection‐1 (FX model) and injection‐2 (2SX model) parameter estimates (*p* = 0.36 and *p* = 0.08, respectively; right panels of Figures 3 and 4).

**TABLE 2:** Parameter estimates obtained from the DCE‐MRI data (acquired at the baseline visit only) following injection‐1 and injection‐2 using both a fast exchange (FX) and a two‐site exchange (2SX) model.

	Injection‐1		Injection‐2	
	FX	2SX	FX	2SX
MBF (mL/min/mL)	0.76 ± 0.17	0.80 ± 0.17	0.68 (0.38–1.32)	0.69 (0.38–1.48)
ECV (%)	19.6 ± 3.1	20.7 ± 3.5	17.6 ± 2.6	18.7 ± 3.0
τ_ic_ (s)	—	0.003 (0–8771)	—	0.00 (0–0.429)

*Note:* Results presented as mean ± SD, when the data were normally distributed or median (range), when they were not.

**FIGURE 3:**
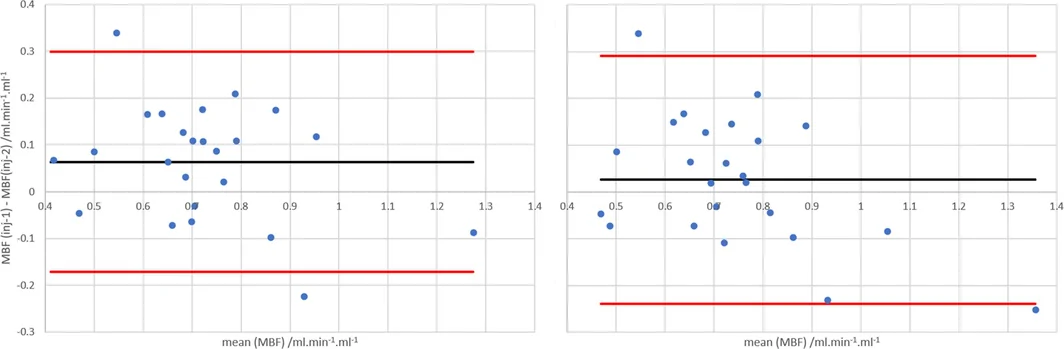
Bland–Altman plots comparing MBF estimated from injection‐1 and injection‐2 using (left) an FX model for both injections, and (right) an FX model for injection‐1 and a 2SX model for injection‐2. The black line represents the mean difference between MBF estimates and the red lines represent the 95% limits of agreement. The *y*‐axis shows injection‐1 MBF minus injection‐2 MBF.

**FIGURE 4:**
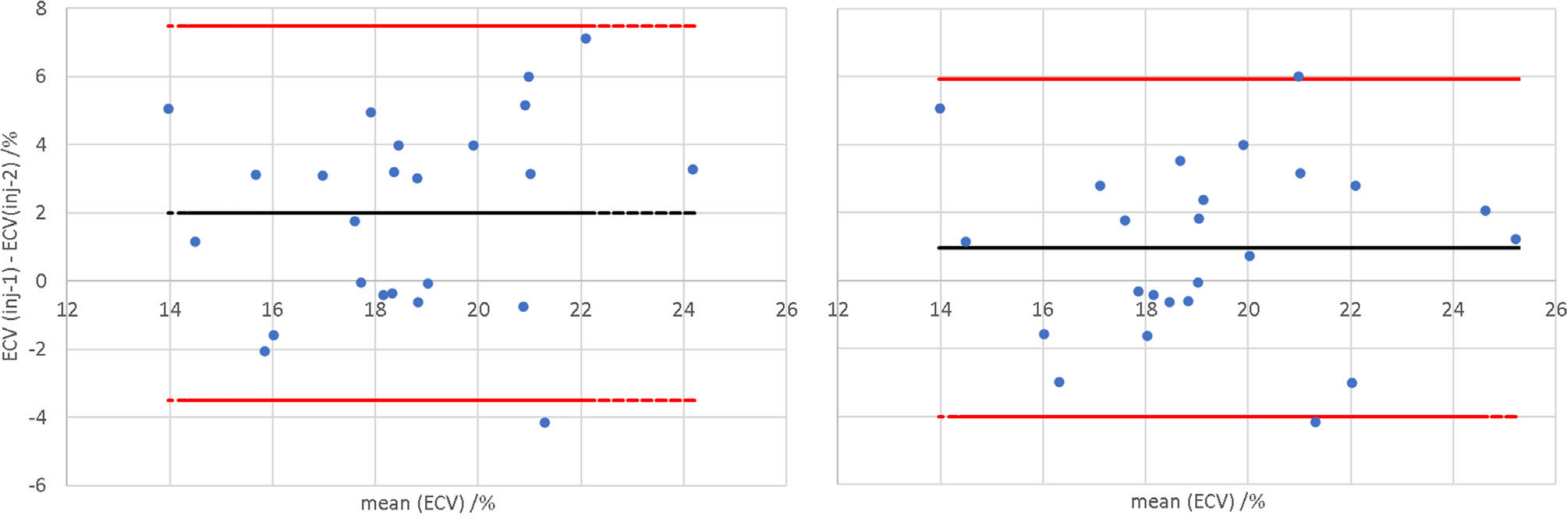
Bland–Altman plots comparing ECV(DCE) estimated from injection‐1 and injection‐2 using (left) an FX model for both injections, and (right) an FX model for injection‐1 and a 2SX model for injection‐2. The black line represents the mean difference between ECV estimates and the red lines represent the 95% limits of agreement. The y‐axis shows injection‐1 ECV minus injection‐2 ECV.

The injection‐1 data were not expected to exhibit sensitivity to WX and the MBF and ECV estimates obtained using the 2SX model were predicted to agree with those obtained using the FX model. However, when the injection‐1 data were analyzed using a 2SX model, like the injection‐2 data, the parameter estimates increased significantly (*p* = 0.002 and *p* = 0.004, respectively). Estimates of τ_ic_ obtained using the 2SX model from the DCE‐MRI data from both injections were hugely variable. Half of the estimates were 0 and the others ranged from values similar to those measured using T1 maps [5] up to almost 9000 s (Table 2). There was no correlation between the estimates of τ_ic_ made at injection‐1 and those made at injection‐2.

The model fits were compared using *F* tests which showed the 2SX model better described the DCE‐MRI data in only 3 of 25 injection‐1 data sets and 2 of 24 injection‐2 data sets.

### 3.3 Comparison of ECV Estimates Using T1 Maps and DCE‐MRI Data

ECV was estimated before and after AVR and at each visit we report three different estimates: using the T1 maps with an LM (ECV(LM)), the T1 maps and an SS model (ECV(SS)), and using the DCE‐MRI data (ECV(DCE)) following injection‐1 with an FX model. The injection‐2 DCE‐MRI data and results obtained using a 2SX model were not considered further (see Section 4).

At baseline (*n* = 25) the average ECV(LM) estimate was 21.7% ± 3.1%, the average ECV(SS) estimate was 27.2% ± 4.5%, and the average ECV(DCE) estimate was 19.6% ± 3.1%. There was a statistically significant difference between the ECV estimates made using both T1 map methods and those made using the DCE‐MRI data (*p* = 0.0024 and *p* < 0.001, respectively).

Post‐AVR (*n* = 20) the average ECV(LM) estimate was 28.2% ± 4.5%, the average ECV(SS) estimate was 31.8% ± 3.7%, and the average ECV(DCE) estimate was 21.9% ± 3.4%. Again, there was a statistically significant difference between the ECV estimates made using both T1 map methods and those made using the DCE‐MRI data (*p* < 0.001 for both).

As noted previously [18], the SS model estimates of ECV were significantly higher than those made using the LM at both visits and both models showed a significant increase in ECV post‐AVR. The DCE‐MRI data highlighted the same patterns, but the absolute values of ECV at each visit and the change in ECV between visits were all lower (see Table 3).

**TABLE 3:** Mean ECV estimates (± SD) and the change in those estimates made using T1 maps (linear model [LM] and shutter‐speed model [SS]) and DCE‐MRI data at baseline and 6 months after surgical aortic valve replacement (post‐AVR).

	Baseline (*n =* 25)	Post‐AVR (*n =* 20)	ΔECV (*n =* 20)
ECV(LM)	21.7% ± 3.1%	28.2% ± 4.5%	6.3% ± 4.5%
ECV(SS)	27.2% ± 4.5%	31.8% ± 3.7%	4.3% ± 6.0%
ECV(DCE)	19.6% ± 3.1%	21.9% ± 3.4%	2.5% ± 3.7%

## 4 Discussion

We hypothesized that a high dose of contrast agent would increase the impact of WX on estimates of MBF and ECV made using DCE‐MRI. There were significant decreases in estimates of both parameters following the high‐dose injection (cumulative dose of 0.15 mmol/kg). Moreover, when the data from the high dose experiment were analyzed using a 2SX model, accounting for WX, average estimates of MBF and ECV increased, and while they remained lower, they were no longer significantly different from the FX model low‐dose estimates. These results support the hypothesis that our high dose DCE‐MRI data were significantly affected by WX.

However, when the low‐dose data were similarly analyzed using a 2SX model, average estimates of MBF and ECV also increased, despite the expected WX insensitivity of those data. An increase in estimates of MBF and ECV obtained using a 2SX model over those obtained using an FX model is almost inevitable: The 2SX model cannot produce decreased estimates of MBF and ECV (this could only happen if τ_ic_ < 0 then the model would be non‐physiological). If estimates of τ_ic_ = 0 then the 2SX model collapses to the FX model and estimates of MBF and ECV remain unchanged, a result seen in half the injection‐1 and injection‐2 data sets. If estimates of τ_ic_ > 0 (which would occur as a result of limited WX but might also occur due to some structured noise in the data or be caused by an error in the assumption of a single compartment tracer kinetic model [14]) then MBF and ECV will increase, a result seen in the remaining data sets. If half the MBF and ECV estimates remain the same and half increase, then this leads to an average increase in MBF and ECV across the sample (injection‐1 and injection‐2). But the addition of τ_ic_ to the model produces only a subtle effect on the fit in most cases (e.g., see Figure 2), hence, when the model fits were compared using an *F*‐test, the simpler FX model was preferred in 44 of 49 cases; estimates of τ_ic_ were hugely variable, as seen before [14, 15] and showed no correlation at all with estimates of τ_ic_ obtained from the T1 data [5]. Taken together, these results suggest that our DCE‐MRI data are insensitive to WX.

If we use our injection‐1 and injection‐2 DCE‐MRI data to test repeatability of the perfusion measures then we obtain excellent within‐subject coefficients of variation (wCV), 8.3% for MBF and 7.6% for ECV. Our repeatability is comparable to that measured at rest in healthy volunteers by Brown et al. [26] in their study using repeat low‐dose injections and the same dual‐sequence DCE‐MRI acquisition used in the current study [20]. Moreover, like us, Brown et al. [26] measured a small but significant decrease in estimates of MBF after repeated contrast agent injections. They attributed this to physiological variation caused by a reduction in subject anxiety over the course of the examination rather than an effect of increasing contrast agent load (and thus increased WX sensitivity). They did not see a reduction in MBF following repeated stress exams where maximal vasodilation was induced (thus no physiological variation), but the same increase in contrast agent load was used.

Given the WX insensitivity of our DCE‐MRI data and the results of the *F*‐tests comparing FX and 2SX models, further assessment of the ECV data was restricted to injection‐1 and an FX analysis. ECV estimates from the DCE‐MRI data at baseline were significantly lower than estimates made using T1 maps (whether analyzed using a LM or SS model) and while the ECV(DCE) estimates showed a significant increase post‐AVR, both the ECV estimates and the change in ECV measured following AVR remained below those estimated using T1 maps (Table 3). These findings do not match those reported by Kunze et al. [13] who showed negligible difference between estimates of ECV made using DCE‐MRI and T1 mapping. However, Kunze et al. conceded that their DCE‐MRI estimates of ECV represented an “upper limit” (due to difficulties measuring an AIF) and their T1 map analysis did not account for WX despite their use of a high dose of contrast agent (0.25 mmol/kg gadopentetate dimeglumine).

Our measures of ECV(DCE) were estimated from data acquired for only ∼2 min post‐injection of contrast agent. ECV(LM) is estimated from data acquired 10 min post‐injection and ECV(SS) is estimated from data acquired from between 4 and 30 min post‐injection. Two minutes is little time for the contrast agent to achieve a uniform distribution throughout the extracellular matrix [7] thus it is possible that our estimates of ECV(DCE) are lower because the contrast agent has not distributed as widely in the extracellular matrix as it has when measured in ECV(LM) and ECV(SS).

### 4.1 Limitations

Data from this preliminary study are limited to patients with AS and the results cannot be generalized to other myocardial pathologies without further research. Hct measurements were made 1 day ± 8 weeks from the date of the MRI scan. This may have affected absolute estimates of ECV in some patients but, since it modifies both FX and 2SX results similarly, will have minimal impact on the sensitivity to WX. We have not investigated changes to the DCE‐MRI pulse sequence that might optimize it to WX [27]. A single compartment model is an over‐simplification of the distribution of contrast agent in the myocardium but provides a balance between accuracy and precision, particularly for the heart at rest where the capillary wall is very permeable to the contrast agent [12]. We have only considered WX across the cell wall in the current study. WX across the capillary wall (transendothelial exchange) is unlikely to be fast [1] though the vascular‐suppressing effects of this on our DCE‐MRI data will lessen the impact of any errors associated with use of a single compartment model to describe contrast agent kinetics.

## 5 Conclusion

DCE‐MRI showed negligible sensitivity to WX even at a high contrast agent dose, suggesting that those performing quantitative rest perfusion studies at low dose may not need to worry about its effects. DCE‐MRI was sensitive to changes in ECV following AVR surgery. However, the estimates of ECV and the change in ECV were both lower than those made using T1‐maps, and further work is needed to investigate these differences.

## Funding

The study was funded by the British Heart Foundation (award PG/20/10008). S.P. is supported by a British Heart Foundation Chair (CH/16/2/32089).

## Conflicts of Interest

Peter Kellman is an independent consultant for Siemens Healthineers & Microsoft Research but has received no direct support for any aspects of this work. The remaining authors declare no conflicts of interest.

## Data Availability

The datasets used and analyzed during the current study are available from the corresponding author on reasonable request.
